# Patients’ perception of the practice of anaesthesia in a teaching hospital in Northern Jordan: a survey

**DOI:** 10.1186/s12871-020-01193-6

**Published:** 2020-11-02

**Authors:** Adel M. Bataineh, Ibraheem Y. Qudaisat, Khaled El-Radaideh, Rawand A. Alzoubi, Mohammad I. Abu-Shehab

**Affiliations:** 1 Department of Anesthesia and Recovery, Faculty of Medicine, Jordan University of Science and Technology, Irbid, Jordan; 2 Department of Anaesthesia and Recovery, King AbduLLAH-I University Hospital, Irbid, Jordan; 3 Department of Anesthesia and Intensive Care, School of Medicine, University of Jordan, Amman, Jordan

**Keywords:** Anaesthesia, Anaesthetist roles, Perception, Jordan

## Abstract

**Background and aim:**

Despite big leaps of progress in its scope, the practice of anesthesia is still suffering from poor public image, especially in developing countries. Little research investigated the public awareness of anesthesia in the Middle East. This study aimed to examine the perception of the practice of anaesthesia among Jordanian patients.

**Methods:**

A standard questionnaire with 29 questions was administered through personal interview to consenting patients. Questions tested patients’ correct knowledge of the identity of anesthetists, their roles and scope of their practice. Awareness was measured using the frequency of correct answers to each survey question. A total awareness score was calculated as the percentage ratio of the number of correct answers to the total number of questions. We classified this score into: Poor< 50%. Moderate 50–75%, and Good > 75% to reflect patient’s overall perception of anesthetists and their roles. Effects of demographic variables on results were also investigated. Appropriate statistical tests were used to summarize and compare results. A total of 513 patients admitted for elective surgery were sequentially approached for enrolment.

**Results:**

Five hundred and five patients were enrolled. Most patients identified anesthesia as a separate practice from surgery (86%). The anaesthetist was identified as a physician by only 37% of patients. Equal importance to both anaesthetists and surgeons was assumed by 71.5% of patents. Only 15% of patients showed good level of total knowledge of anaesthetist roles, while 51% scored poorly. Highest awareness was of anaesthetist’s preoperative roles (65.1%). Age was the only demographic factor affecting studied awareness (*P = 0.009*).

**Conclusion:**

Although the importance of anesthetist is well perceived among Jordanian patients, there is still some ignorance in their knowledge of the details of anesthesia practice. Active communication efforts and patient education by anesthetists are needed to improve the public status of the specialty.

**Supplementary Information:**

The online version contains supplementary material available at 10.1186/s12871-020-01193-6.

## Background

Since its early beginnings in the nineteenth century, the practice of anesthesia had been in a state of continuous evolution [1]. This evolution culminated in the announcement of anesthesia as a specialty in 1939 during the New York World Fair [2]. Its scope of medical care had extended beyond the perioperative period to include more and more medical services. In addition to the perioperative anesthetic care, the practice now covers acute and chronic pain management, intensive care units, and resuscitation care services. This is in addition to the well-established role of anaesthetists in undergraduate medical education [3]. Consequently, the role of the anaesthetist, as a medical care giver, has grown tremendously and extended beyond the boundaries of operating theatres.

Unfortunately, the status of anesthesia is still underestimated within medical specialties, and its role misunderstood among lay communities who are not trained, qualified or experienced in medical field [4]. Even in the developed world, the progress of this understanding was slow and is still incomplete, except for few countries like Switzerland [4, 5]. The perception of anesthesia and role of anaesthetist in the developing countries is still lagging behind that in the developed world [6].

Anesthetic care in Jordan is currently provided by a qualified anesthesia specialist doctor assisted by an anesthesia technician who is a graduate from a two-year study program from a community college. The aim of our study was to investigate the state of perception of anaesthesia and role of anaesthetist among Jordanian patients admitted for elective surgery in a teaching hospital in the north of Jordan.

## Methods

### Study design, settings and participants

A cross-sectional questionnaire-based opinion survey was carried out through personal interviews at King Abdullah University Hospital, Al Ramtha-Jordan, in the period between March and June 2019. After obtaining approval from the research committee of the faculty of medicine at Jordan University of Science and Technology, and hospital’s IRB committee, 18-year-old and above patients, admitted for elective surgery the next day in different surgical specialties, were approached in their wards for participation in the survey. Exclusion criteria included patients who were mentally incapacitated, physically or emotionally unstable, patient with pain score ≥ 4 on the visual analogue scale, and those receiving any medications that can alter their decision making. The study was performed following the guidelines of the Declaration of Helsinki [7].

### Questionnaire

After a review of previous similar literature, a questionnaire with a total of 29 questions was developed and structured by the first two authors to cover the intended study objectives. It was then reviewed by the other three researchers. It consisted of three parts: the first part was of demographic data including previous surgery, the second part contained questions about patients’ perception of anaesthetists in terms of their qualifications (i.e. fulfilling the official requirements for a specific practice in the medical field), and importance (how much their role counts in maintaining the wellbeing of the patient in the perioperative period), and the last part examined patients’ awareness of the roles of anaesthetist both inside and outside theatre, (Additional file 1). The survey questionnaire was administered through personal interviews in the commonly spoken (colloquial), non-formal Arabic language. It was first piloted to 10 patients, and necessary modifications in questions were made before embarking on the survey proper. Responses from each patient were recorded on a separate form, given a serial number, and the patient’s identity was kept anonymous. After completion each interview, the data was entered into an Excel database file for future processing and analysis.

### Study outcomes

The primary outcome of this study was to determine patients’ knowledge and perceptions of anesthesia and anaesthetists. In order to achieve that, we used the frequencies of correct responses to the different relative questions. Also, we calculated a knowledge percentage score of anaesthetist roles as the percentage of correct answers out of responses to all 19 questions. A similar score was also calculated for each stage of perioperative management and for the roles of anaesthetist outside the operating theatre. We classified the knowledge percentage score scale into poor, moderate, and good as follows: Poor< 50%. Moderate 50–75%, and Good > 75%. The secondary outcome was to assess the effect of demographic factors on this perception and knowledge.

## Statistics

Statistical Analysis was carried out using SPSS version 22 software (IBM, Chicago, IL, USA). Descriptive statistics were used to summarize data using frequencies for nominal data and mean (±SD) for continuous variables. For secondary study outcomes, chi-square test and independent samples t-test were used as appropriate for comparisons between demographic subgroups.

## Results

Five hundred and thirteen patients were sequentially approached for participation in the survey. Eight patients declined participation, and 505 patients agreed to be enrolled, giving a response rate of 98%. The mean age of participants was 43.5 (14.9±) years, and 276 (55%) were females. Most patients (92%) were city dwellers, and (44%) had education higher than high school. Two hundred and sixty-three patients (52%) had previous surgeries, 39% of them were in teaching hospitals. All enrolled patients completed the survey without withdrawals. Table 1 summarizes the details of demographic variables.

**Table 1:** Demographic data of study participants

Variable			***n***	%
GENDER		Male	229	45.3
		Female	276	54.7
AGE GROUP		< 60 yr	426	84.4
		> = 60 yr	79	15.6
EDUCATION		up to High school	281	55.6
		Higher education	224	44.4
RESIDENCE		City	332	65.7
		other	173	34.3
PREVIOUS SURGERY	Yes	Teaching hospital	197	39
		other hospital	166	32.9
		Total	363	71.9
	No		142	28.1

**Fig. 1:**
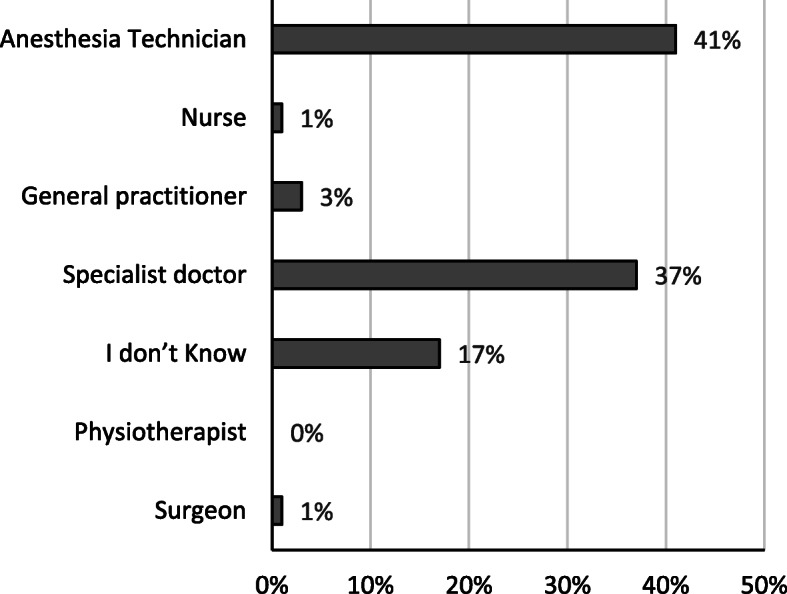
Frequencies of patients’ perception of anaesthetist qualification

**Fig. 2:**
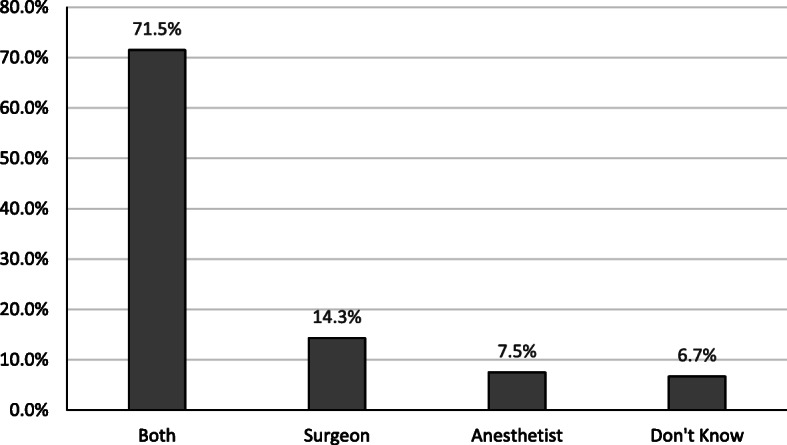
Patients’ perception of the more important doctor in the operating theatre

Only 37% of patients were aware that the one who administers anesthesia is a fully qualified physician, compared to 41% who believed that a technician does the job. However, 17% did not know what to answer, with negligible percentages of patients assuming the job done by other professions, Fig. 1. Most of our patients (83%), identified the anaesthetist as a separate practitioner from the surgical team, and 86% knew that he does not leave the theatre during surgery. About 71.5% of patients in our study considered the roles of both anesthetists and surgeons equally important in maintaining patients’ well-being in the perioperative period, Fig. 2.

Table 2 summarizes the frequencies of patients’ correct perception of the perioperative roles of the anaesthetist. Preoperative evaluation of patients including ordering investigations and asking for necessary consultations was recognized as an anaesthetist role by about half of the participants. Two-thirds of patients were aware that the decisions on fitness for surgery under anesthesia and the required fasting time were also roles of the anaesthetist. A higher proportion of about three-quarters of patients were aware of the role of anaesthetist in obtaining informed consent from each patient before anesthesia.

**Table 2:** Frequencies of patients’ correct answers to knowledge questions about the roles of anaesthetists

Role		Correct answer	
		*No.*	%
Pre-operative Roles	Preoperative assessment of patients	**266**	**52.7**
	Asking for investigations and consultations	**279**	**55.2**
	Deciding on patient fitness to undergo surgery	**332**	**65.7**
	Dermining required fasting duration	**305**	**60.4**
	Expalining anesthetic details before operation	**388**	**76.8**
	Answering all patient questions about anesthesia	**402**	**79.6**
Intra-operative Roles	Giving patient the required hypnotic drugs	**380**	**75.2**
	Giving patients the required analgesic drugs	**186**	**36.8**
	Administering any medications that the patient may need in OR	**153**	**30.3**
	Continuously monitoring the patient without leaving the OR	**368**	**72.9**
	looking after patients needs of fluids	**156**	**30.9**
	Estimating blood loss and carrying out necessary blood TX	**112**	**22.2**
	Waking patient up in the operting room at the end of surgery	**363**	**71.9**
Postoperative Roles	Escorting patient to the recovery room	**268**	**53.1**
	Superising patient in the recovery room	**279**	**55.2**
	Treating any complications in the recovery room	**161**	**31.9**
Roles outside operating theater	Managing patients in the intensive care unit	**128**	**25.3**
	Doing cardiopulmonary resuscitation	**202**	**40.0**
	Chronic and acute pain management	**143**	**28.3**

Patients’ awareness of anaesthetist’s intraoperative roles was not uniform. It was most apparent for putting patients to sleep (75%), monitoring them throughout surgery (73%), and waking them up at the end (72%). Recognition of other roles was only observed in about one-third of patients for the administration of analgesic drugs, other medications, and fluid needs. Blood transfusion was believed to be an anaesthetist role by only 22% of patients.

Escorting patients to the post-anesthesia care unit (PACU), and following them up there, were believed to be roles of the anaesthetist by a little more than half of patients. Surprisingly, only 32% of our patients considered the management of postoperative medical complications in PACU an anaesthetist job.

Most patients were not aware of the roles of anaesthetists outside the operating theatres. Awareness was highest for cardiopulmonary resuscitation (40%), followed by the management of acute and chronic pain (28%). Awareness of the role of looking after intensive care patients was observed only in 25% of responses.

Almost half of our patients (51%) showed poor level of knowledge of anaesthetists’ roles. Good level of knowledge was shown by only 15% and moderate level by the remaining 34% of participants. Age was the only demographic finding that had significant effect on the level of awareness among patients of the roles of anaesthetists Table 3. Older patients (> 60 yr) were significantly more aware of the roles of anaesthetists compared to younger ones (*P* = 0.009). The genders had almost the same level of awareness while higher education, city-dwelling, previous surgery, and teaching hospital surgical experience had a minimal positive effect that did not reach statistical significance.

**Table 3:** Patients’ knowledge score of the anaesthetist’s roles at different points of care

Knowledge Score	Mean
All roles	**50.8 (23.1)**
Preoperative roles	**65.1 (28.7)**
Intraoperative roles	**48.6 (25.5)**
Postoperative roles	**46.7 (37.9)**
Roles outside operating theaters	**31.2 (34.6)**

On the other hand, when we calculated the average score for each stage of perioperative care separately, the preoperative roles of the anaesthetist turned out to be enjoying the highest level of awareness among patients (65%). Nonetheless, this score was still in the moderate level of knowledge. The level of awareness of anaesthetists’ roles in other stages of patients’ care were unfortunately poor, Table 4.

**Table 4:** Effects of demographic factors on level of patients’ knowledge of anaesthetists’ roles

Demographic variable	Subgroup	n.	Knowledge percentage Score (%)	***P value***
Gender	M	229	50.77 (23.1)	0.977
	F	276	50.76 (23.2)	
Age group	< 60 yr	426	49.6 (23.1)	0.006
	> = 60	79	57.3 (22.2)	
Education group	school	363	49.7 (22.2)	0.325
	> school	142	51.9 (23.7)	
Residence	City	332	52.0 (23.2)	0.093
	other	173	48.4 (22.8)	
Previous surgery	Yes	363	51.4 (23.0)	0.304
	No	142	49.1 (23.5)	
Previous surgery Hospital	Teaching	197	49.8 (25.8)	0.353
	non-teaching	166	47.3 (24.8)	

## Discussion

The results of this survey of Jordanian patients admitted for elective surgery in a teaching hospital about their perception of anaesthetists and their roles, showed a mixture of appreciation and misconception. Patients were aware of the standalone nature of anesthetic practice from the surgical team and were also informed that the anaesthetist is committed to his patient in the operating theatre. Furthermore, the importance assumed by most of our patients to the role of anaesthetists was equal to that of surgeon by 71.5% of them. However, only 37% of them recognized the anaesthetist as a specialized doctor. Furthermore, the anaesthetist was thought to be a technician by an even higher percentage of patients (41%). This misconception may be because anesthetic services in Jordan used to be provided mainly by anesthesia technicians in the past. The scarcity of anaesthetists in the country mandated this level of qualifications until the mid-eighties of the last century. At that time, regulations were set by the ministry of health, which limited the provision of anesthetic care to physician anaesthetists. Unfortunately, there are no previous national baseline values to compare our results with. However, it is apparent, after about three and half decades, that the rise in the public awareness of the professional identity of the anaesthetist is slow. This slow progress of awareness about anaesthetists was observed even in developed countries at some point and reflected deficient perioperative teaching and communication on the side of anaesthetists about their practice [4].

Our patients’ awareness of the different roles of the anaesthetist is consistent with their perception of his qualifications. This consistency was most apparent in the awareness results of the intraoperative roles of the anaesthetist, Table 2. Here, patients voted most for the roles that are mainly concerned with the sleep process, while more medical roles, like fluid management, blood transfusion, and administration of other necessary drugs, were perceived only poorly. On the other hand, the medical roles of preoperative patient assessment and postoperative management of complications were also considered least by patients to be roles of anaesthetists. Our results about the physician status of anaesthetists are close to those reported in previous studies from different countries in the developing world [8–11].

Compared to other perioperative and outside-theatre roles, the preoperative duties of anaesthetists were the most recognized duties by our patients, with a knowledge score of 65.1%, Table 3. A possible explanation could be that the patient’s knowledge about the roles of anaesthetists was likely obtained by witnessing rather than education. The total loss of consciousness during general anesthesia and the residual effects of anesthetic drugs in the recovery room will deprive patients of this resource of learning.

Age was the only demographic factor significantly affecting our patients’ level of awareness of the roles of anaesthetists, Table 4. This finding is intriguing as older patients are more familiar with the older system of anaesthetist qualifications and roles. However, once again, if the practice of anesthesia was not brought to patient’s awareness through ongoing education and proper role modelling, patients will end up with the implicit type of learning through exposure to the different sources of information like friends, media, and observation during previous surgical experiences. This implicit type of learning is believed to be preserved with healthy aging, and older patients will have the privilege of higher exposure to information through this type of learning [12]. The active role of educating patients about this specialty seems to be deficient so far among anaesthetists in our country and unfortunately even in teaching hospitals.

Regardless of their level of education, there is always a desire amongst patients to know more about their management, and efforts in this regard are mandatory to increase awareness of the specialty [13, 14]. The benefits of educating patients about anesthesia and what to expect from their anaesthetists are multiple. Patients’ fear from the loss of control over their own bodies during anesthesia can negatively affect their perioperative course of management to the extent that they may choose to cancel their surgeries. Enlightening patients with the details of their perioperative anesthetic care in terms of personnel and practice, will alleviate much of their anxiety and make them feel better in control over this part of their hospital experience [15, 16]. On the other hand, anaesthetists will be able to build rapport with their patients during their preoperative physician-patient encounters [4, 17]. This rapport fosters the trust and confidence of patients in their anaesthetists and will help improve the poor public image of the practice of anesthesia and even reduce incidence of malpractice litigations [4, 18]. The patients’ perception and knowledge of the practice of anesthesia was shown to be improved by active patient education endeavors [15]. This education can take multiple forms which include: preoperative assessment clinics, informative preoperative visits, use of educational videos, use of public media, educating other health professionals about anesthesia, and getting anaesthetists involved in administrative hospital committees [4, 19–21].

Our data collection and analysis were completed before the start of the Coronavirus pandemic. However, in their justification for the country’s lockdown, the national Coronavirus committee raised the concern that the health system may be overwhelmed by the probable large numbers of critical Covid-19 victims. Media discussions about the adequacy of the country’s repository of beds, ventilators, and staff followed. These events shed a good light on the importance of anesthetists. So, if our study was to be repeated nowadays, we expect that it will reveal positive changes in the results especially those of anesthetist identity and the critical care part of the practice. Results about anesthetist’s perioperative roles are expected to minimally change.

Our survey was carried out in the northern part of Jordan, and our results may have changed had the data been collected from other country regions too. Another limitation was the unfortunate lack of external peer-reviewing of our study questionnaire, which could have increased confidence in its effectiveness.

Our study results gave an idea about the current state of patients’ perception of anesthesia and anaesthetists in Jordan. We believe that results represent an addition to the medical literature from this part of the world in this field and can be used as a reference for any future studies in this regard.

## Conclusion

Our study showed that, although patients in Jordan are aware of the importance of anesthetists in maintaining their well-being during perioperative care, there is still ignorance in their knowledge about the details of their practice. Efforts to educate patients about anesthesia in terms of practice and personnel are needed to improve the current state of perception to meet international standards.

The leading role of intensive care practitioners, including anesthetists during the recent pandemic of the Coronavirus, may have played a role in improving the public’s perception of this particular role of anesthetists.

## Supplementary Information

**Additional file 1: Appendix 1:** Survey Questionnaire

## Data Availability

All data generated or analysed during this study are included in this published article.

## Abbreviation

PACU: Post-Anaesthesia Care Unit
