# Supplementary material for: Patients’ perception of the practice of anaesthesia in a teaching hospital in Northern Jordan: a survey
Source: BMC Anesthesiol. 2020 Nov 2;20:276. doi: 10.1186/s12871-020-01193-6 (PMC7604332; doi:10.1186/s12871-020-01193-6)
Supplement: Supplementary file 1 — Additional file 1: Appendix 1: Survey Questionnaire [file 12871_2020_1193_MOESM1_ESM.docx]

**Appendix: Patient questionnaire**

***Part 0ne: Demographic information***

1: - Age: ………………….

2: - Gender: 🗆 Male 🗆 Female

3: - Education: 🗆 Illiterate 🗆 School 🗆 High education

4: - Residence: 🗆 City 🗆 Other

5: - Intended operation: ……………………………….

6: - Previous operations: 🗆 Yes 🗆 No

If Yes, where: …………………… Type of anesthesia: 🗆 General Anesthesia 🗆 regional

***Part Two: Anesthesia Provider***

1: - The anesthetist is a:

🗆 member of the surgical team 🗆 Head of special team.

2: - The anesthetist role requires staying in theatre throughout surgery: 🗆 Yes 🗆 No

3: - The anesthetist is a qualified:

🗆 Technician 🗆Nurse 🗆physiotherapist 🗆General practitioner

🗆 Specialist physician 🗆Surgeon 🗆 I don’t know.

4: - The one with more important role during operation is:

🗆 Surgeon 🗆 Anesthetist 🗆 Both 🗆 I don’t know

***Part three: Roles of anesthesia provider:***

| **Role** | | **Agree ?** | |
| --- | --- | --- | --- |
|  |  | **Yes** | **No** |
| **Preoperative roles** | 1: Preoperative assessment of patients | 🗆 | 🗆 |
|  | 2: Asking for investigations and consultations | 🗆 | 🗆 |
|  | 3: Deciding on patient fitness to undergo surgery | 🗆 | 🗆 |
|  | 4: Demining fasting duration | 🗆 | 🗆 |
|  | 5: Explaining anesthetic details before operation | 🗆 | 🗆 |
|  | 6: Answering all patient questions about anesthesia | 🗆 | 🗆 |
| **Intraoperative roles** | 1: Giving patient the required hypnotic drugs | 🗆 | 🗆 |
|  | 2: Giving patients the required analgesic drugs | 🗆 | 🗆 |
|  | 3: Administering any medications that the patient may need in Operating theater | 🗆 | 🗆 |
|  | 4: continuously monitoring the patient without leaving the OR | 🗆 | 🗆 |
|  | 5: Looking after patients’ needs of fluids | 🗆 | 🗆 |
|  | 6: Estimating blood loss and carrying out necessary blood transfusion | 🗆 | 🗆 |
|  | 7: Waking patient up in the operating room at the end of surgery | 🗆 | 🗆 |
| **Postoperative roles** | 1: Escorting patient to the recovery room | 🗆 | 🗆 |
|  | 2: Supervising patient in the recovery room | 🗆 | 🗆 |
|  | 3: Treating any complications in the recovery room | 🗆 | 🗆 |
| **Outside-theater roles** | 1: Managing patients in the intensive care unit | 🗆 | 🗆 |
|  | 2: Doing cardiopulmonary resuscitation | 🗆 | 🗆 |
|  | 3: Chronic and acute pain management | 🗆 | 🗆 |
